# The efficacy and safety of acupuncture for perimenopause symptom compared with different sham acupuncture control groups: A protocol of systematic review and meta-analysis

**DOI:** 10.1097/MD.0000000000019366

**Published:** 2020-03-06

**Authors:** Qiujun He, Yajing Ren, Yanqiu Wang, Feng Zhang, Sanyin Zhang

**Affiliations:** a College of Basic Medicine, Chengdu University of Traditional Chinese Medicine; b Chengdu University of Traditional Chinese Medicine; c Chengdu Fifth People's Hospital; d College of Acupuncture and Tuina, Chengdu University of Traditional Chinese Medicine, Chengdu, Sichuan, China.

**Keywords:** acupuncture, meta-analysis, perimenopausal symptoms, sham acupuncture control

## Abstract

**Background:**

Perimenopause is a period that every woman must go through, most people are more or less affected by perimenopausal symptoms, it to affect women's health, work, life, and economy. As acupuncture treatment is more and more increasing in perimenopausal symptoms, there have also been many clinical trials about it. But the results of the trials are inconsistent. Therefore, we will conduct a systematic review and meta-analysis of the safety and efficacy of perimenopausal symptoms treated with acupuncture.

**Methods:**

The protocol followed Preferred Reporting Items for Systematic Reviews and Meta-Analyses Protocols. RCT study on different acupuncture interventions for perimenopausal symptoms will be searched in 8 databases (PubMed, EMBASE, the Cochrane Library, the web of science, CBM, CNKI, WAN FANG, and VIP). Besides, the search will also be performed on the clinical trial research platform if necessary. The primary outcome that will be extracted: the Flushes per 24 hours, the Frequency of hot flashes, the severity of hot flashes, the menopause-related symptom score, the treatment efficacy, the adverse event. Endnote software X8 will be used for study selection, STATA 13.0 and Review Manager software 5.3 will be used for analysis and synthesis. These studies selection, data extraction, and risk of bias assessment will be conducted by 2 independent reviewers.

**Results:**

This study will provide the results: 1. the primary and secondary outcome indicators of different acupuncture intervention measures (traditional hand acupuncture, moxibustion, ear acupuncture, laser, acupressure points) for perimenopausal symptoms. 2. The effects of different control groups (medicine control, routine care, waiting, and sham acupuncture control) on the analysis results will be reported, especially the effects of different sham acupuncture control (invasive/noninvasive) on the analysis results.

**Conclusion:**

This systematic review and meta-analysis study hopes to provide useful evidence for better use of different types of acupuncture in treat perimenopausal symptoms and better design of control groups in related clinical trials. In addition, the research conclusion will be published in peer journals.

**OSF REGISTRATION NUMBER** DOI 10.17605/OSF.IO/VZCKU Ethics and dissemination This conclusion of the study will be published in peer journals. The ethical approval is not required because there is no direct involvement of human.

## 1 Introduction

Perimenopause syndrome, referred also as a climacteric syndrome. The perimenopausal symptoms include premenopause, menopause and postmenopause. Perimenopause is a normal and important aging phenomenon that occurs in women's life. Although some women have no obvious symptoms during this period, Most women still suffer from perimenopausal symptoms that affect health, work, life, and economic status.^[1–8]^ The most common and frequent episode of perimenopausal symptoms is hot flashes,^[2,9]^ which affect around 75% of menopausal women,^[10–13]^ In addition, the incidence of menopause insomnia is as high as 39% to 47%,^[14,15]^ Other symptoms include anxiety, fatigue, irritability, weight gain, night sweats, vaginal dryness, and urinary incontinence.^[16–19]^ These perimenopausal symptoms begin 1 to 2 years before menopause and may persist from 6 months to more than 10 years.^[2,20]^ These menopause symptoms may be caused by the perimenopause itself, or it may be a chain reaction caused by one of them. These chain reactions may cause or promote the occurrence of diseases such as high blood pressure, diabetes, obesity, cardiovascular diseases, psychological diseases and so on while accelerating aging. Hot flashes can affect women's work and quality of life, Of course, sleep will also be affected.^[3–5,7,8,21]^ In addition, hard to falling sleep has been shown to associate strongly with anxiety,^[22,23]^ Insomnia and anxiety of menopausal women have a chain effect and cause depression.^[24]^ So it can be seen that the symptoms of perimenopause are not single, there may be multiple symptoms. It may even promote or cause other diseases.^[25–27]^

What is the cause of such a wide-ranging, long-lasting disease? The etiology of perimenopausal syndrome, which is now known to be due to the decline or disappearance of ovarian function, hormone levels fluctuations, and instability.^[16,17,28,29]^ Therefore, hormone replacement therapy has become the most effective treatment for the perimenopausal syndrome.^[13,17,30–32]^ But the Women's Health Initiative (WHI) reported an increase in cases of Cardiovascular disease after hormone therapy.^[33–35]^ Considering the duration of perimenopausal symptoms, hormones are not suitable for long-term use.^[36–39]^ Therefore, more people choose to try non-hormonal replacement therapy,^[40–44]^ acupuncture is one of them.^[45–47]^ Acupuncture treatments include traditional hand acupuncture,^[48]^ electroacupuncture,^[49]^ ear acupuncture,^[50]^ laser acupuncture,^[51]^ acupressure,^[52]^ moxibustion,^[53]^ etc. Research reports that acupuncture can relieve perimenopausal symptoms and improve quality of life.^[45,46,54]^ Although according to research reports that the efficacy of acupuncture has obvious advantages compared with the waiting group^[46]^ or the usual care controls,^[55]^ it also has certain effects compared with western medicine control,^[56]^ it compared with fake acupuncture is highly controversial.^[46,57,58]^ The related meta-analysis studies also showed it.^[59,60]^ A meta-analysis study by Li et al Showed that The number of RCTs that compared the acupuncture control to a sham group is too small, and some studies had a flawed methodology, making it difficult for them to generate reliable conclusions about the efficacy of acupuncture.^[61]^ Therefore, the systematic review and meta-analysis hope to further analyze the efficacy and safety of acupuncture in the treatment of perimenopausal symptoms, focusing on the analysis of different acupuncture interventions and different controls, especially sham acupuncture controls.

## 2 Methods

### 2.1 protocol design

This meta-analysis and systematic review will be based on the guidance of the Preferred Reporting Items for Systematic Reviews and Meta-analysis Protocols (PRISMA-P) for systematic reviews of interventions.^[62,63]^ The ethical approval is not required because there is no direct involvement of human.

### 2.2 Eligibility criteria

#### 2.2.1 Type of participants (P)

The patients in this study will include the woman who transitional period before and after menopause.^[28]^ These women are 40 to 55 years and have perimenopausal symptoms (hot flashes, night sweats, anxiety, palpitations, fatigue, headaches, etc).

#### 2.2.2 Type of interventions (I)

The acupuncture treatment methods of the intervention group included traditional hand acupuncture, electroacupuncture, auricular acupuncture, moxibustion, laser acupuncture, acupressure, etc. There are no acupuncture points, frequencies, and needle retention times.

#### 2.2.3 Type of comparisons (C)

The control group included waiting, routine care, medicine control, Sham acupuncture (invasive/noninvasive), etc. The waiting control and routine care control will not be treated with any acupuncture; the medicine control includes Chinese medicine control and western medicine control; the sham acupuncture control includes non-invasive control (Clinical trial research uses special equipment to prevent the needle from penetrating into the skin but to make patients think they are receiving acupuncture treatment)^[48,58]^ and invasive control (the needle penetrates the skin but does not work. It does not require the feeling of “Deqi” Or acupuncture in places without acupoints).^[64,65]^

#### 2.2.4 Type of outcome measures (O)

##### 2.2.4.1 Primary outcome

(1) The flushes per 24 hours.
(2) The frequency of hot flashes.
(3) The severity of hot flashes.
(4) The menopause-related symptom score (the Kupperman index KI, the Menopause rating scale MRS).
(5) The treatment efficacy.
(6) The adverse event.

##### 2.2.4.2 secondary outcome

(1) Hormone level (FSH, LE, E2).
(2) Menopausal quality of life score (WHOQOL).
(3) Depression scale assessment.
(4) Sleep quality assessment.
(5) The expected value of acupuncture.
(6) Intervention acupuncture experience.

#### 2.2.5 Type of study (S)

The design method is the literature of randomized controlled trials (RCTs), no date or language limits for publication were set.

### 2.3 Exclusion criteria

The following conditions will be excluded:

(1) (a) Received any other alternative therapies 4 weeks before enrollment; (b) ovarian cyst, tumor, oophorectomy, or hysterectomy; (c) severe metabolic, thrombo-embolic or endocrine disease, uncontrolled hypertension or use of sedatives, anxiolytic or antidepressant medication, or use of narcotics.
(2) While this study will exclude any literature regarding acupuncture combined with drugs as the intervention group, as well as the literature that other drugs can be used at the same time during acupuncture treatment.
(3) Non-randomized controlled trials will be excluded.
(4) We will exclude the study that data cannot be extracted, duplicate data, and cannot be provided in full text. However, for studies with insufficient data, we will try to contact the authors of these studies to provide more complete data.

### 2.4 Data source

We will perform a systematic clinical trial research search in databases of PubMed, Embase, Cochrane, Web of Science, CBM, CNKI, WAN FANG, and VIP. If necessary, we will also Search some trial research registration platforms. No date or language limits for publication were set. Search terms for this study include perimenopause, hot flashes, acupuncture, acupressure, electroacupuncture, ear acupuncture, RCTs, etc. The search strategy is designed by the combination of MeSH words, free words and truncation search. The specific search strategies are listed in Table 1.

**Table 1:**
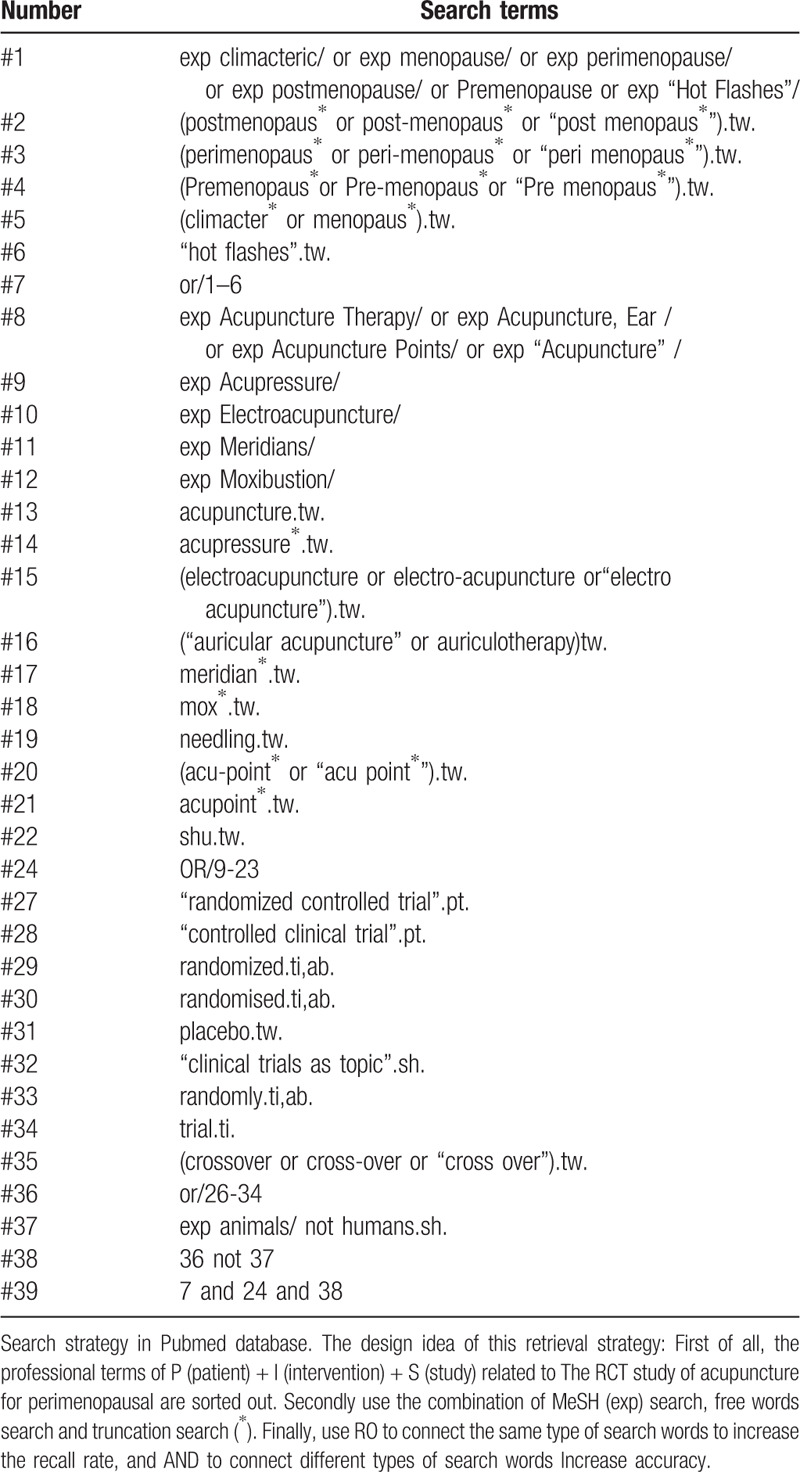
Search strategy in Pubmed database.

### 2.5 Study selection

**Figure 1:**
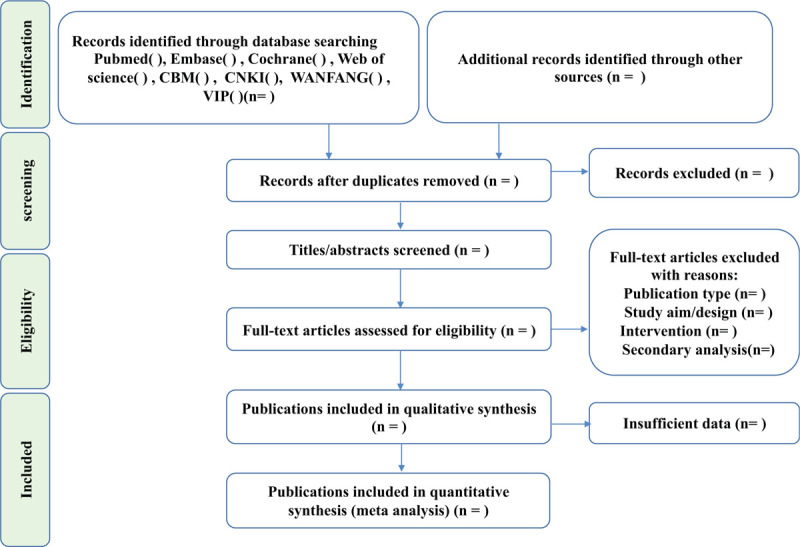
The flow chart of study selection has been designed under the guidance of the Preferred Reporting Items for Systematic Reviews and Meta-analysis Protocols).

All results retrieved from the database will be imported into the EndNote software (X8), and an automatic search for duplicate studies will be set up. After excluding duplicate studies, the 2 authors will independently screen the remaining literature by title and abstract. If the title and abstract include exclusion criteria or do not include inclusion criteria, create a “Title Abstract Exclusion ” in endnote and move the clinical trial study into it. The remaining clinical trials will be searched for full text. Two authors will assess eligibility for the full text of these studies, and create “Publication type” “Study aim/design” “Intervention” “secondary analysis” in endnote according to the reasons for exclusion. The excluded clinical trial studies were moved into the corresponding group according to the reasons for exclusion. Two authors should contact the author of the study if the clinical trial studies are classified as an “insufficient” category due to unclear information or missing data. In addition, all processes will be performed independently by the 2 authors, then discuss the results together. the third review author will identify the study When the opinions of the 2 authors diverged. The select study flowchart Figure 1.

### 2.6 Data extraction

Data will be extracted independently by reviewers using a standardized form. Extracted information included:

(1) Publication features (title, authors, year of publication, journal);
(2) Characteristics of research objects (number of participants, age and sex);
(3) Measures of intervention/control group (Acupuncture type, acupoints, needle retention time, times, etc);
(4) Outcome indicators (For example MRS, hot flashes, menopause index, etc);
(5) The key information of biased risk assessment;
(6) Outcome data will be divided into the pre-treatment data table, the in-treatment data table, and the follow-up data table according to the clinical study time so that the change value can be calculated later. (mean, SD, CI, etc).

### 2.7 Risk assessment

In this study, 2 researchers will conduct risk assessments independently. After the assessment, the results of the cross-check can be discussed and resolved by other researchers and 2 researchers. The final decision will be made by the third author When the 2 authors have a controversy with the assessment. The bias risk assessment included in the study will conduct using the bias risk assessment tool recommended by Cochrane manual version 5.1.0 for RCT. the valuation content includes random method design; assignment concealment; whether to use blind method for subjects and researchers; whether the result data is complete, whether there is a selective report of research results, etc. The rating is divided into high risk, low risk, and unclear. The assessment will be performed using Review Manager software 5.3 software and a risk assessment form will be drawn.

### 2.8 Insufficient data

We will contact the first corresponding authors of the included studies to get missing or insufficient trial data by email. If the data cannot be obtained. We will analyze whether the missing data has an impact on the meta-analysis, and if the impact is large, this clinical trial study will be excluded.

### 2.9 Data analysis

In this study, Revman 5.3 and STATA 13.0 statistical software will use for analysis. If the includes outcome indicator include 10 or more articles, we will use a funnel chart to test the risk of publication bias.

#### 2.9.1 Measures of curative effect

The confidence intervals (CIs) will be set to 95% for both continuous outcomes and dichotomous outcomes. The dichotomous outcomes (the effective rate and adverse events) will analyze the rate ratio. For continuous variables, the mean and SD of the change value were will calculate according to the baseline value and endpoint value of the extracted data. If the included data is not mean and SD, calculate and analyze according to the formula.

#### 2.9.2 Assessment of heterogeneity

The heterogeneity in the study will be analyzed by the *X*^2^ test (the test level was α = 0.1), and evaluate with *I*^2^ statistics. The fixed effects model will be used for the analysis of this outcome indicator, when Its heterogeneity test result is *I*^2^ < 50% (No heterogeneity). if the heterogeneity test result is *I*^2^ ≥ 50% (have heterogeneity), after excluding obvious clinical and methodological heterogeneity, the random-effects model will be used for the analysis of this outcome indicator.

#### 2.9.3 Subgroup analysis

The subgroup analysis is key in this study design.

(1) We will perform subgroup analysis on outcome indicators with different controls. The subgroup analysis of the control group includes waiting, routine care, medicine control, Sham acupuncture (invasive/noninvasive), etc.
(2) We will also perform a subgroup analysis of outcome indicators with different interventions. The subgroup analysis of the intervention group includes ear acupuncture, electroacupuncture, moxibustion, acupressure, traditional hand acupuncture, etc.

#### 2.9.4 Sensitivity analysis

When there is an insufficient sample size, missing data, quality of analysis and research, methodological elements, etc, we will perform sensitivity analysis. The specific implementation is to eliminate the single study, then analyze again, and evaluate the difference between the eliminated results and the original combined results

## 3 Discuss

Hormone replacement therapy has become the most effective treatment for perimenopausal syndrome,^[16,17,28,29]^ but some studies have shown that hormones are not suitable for long-term use.^[36–39]^ Therefore, more people choose to try non-hormonal replacement therapy,^[40–44]^ acupuncture is one of them.^[45–47]^ There are more and more clinical trials of acupuncture treatment for perimenopause, and some meta-analysis researches related to it have also appeared.^[54,59–61]^ These studies demonstrate that acupuncture has a certain effect on one or more of the outcome indicators of perimenopause symptoms compared with the waiting group^[46]^ or the routine care group.^[55]^ But the comparison with sham acupuncture is still controversial.^[57,58]^ At the same time, these studies have analyzed the effects of acupuncture on perimenopausal symptoms, but there is no comprehensive analysis comparing the effects of different acupuncture intervention methods on the outcome indicators of perimenopausal symptoms, which may be the reason for the insufficient sample size at that time.

Therefore, it is necessary to further analyze the safety and efficacy of acupuncture in the treatment of perimenopausal symptoms. Except for routine efficacy and safety analysis, this study focused on subgroup analysis. On the one hand, the subgroup analysis of the control group includes waiting, routine care, medicine control, Sham acupuncture, etc. The sham acupuncture control includes non-invasive control (Clinical trial research uses special equipment to prevent the needle from penetrating the skin but to make patients think they are receiving acupuncture treatment)^[48,58]^ and invasive control (the needle penetrates the skin but does not work. It does not require the feeling of “Deqi” Or acupuncture in places without acupoints).^[64,65]^ On the other hand, intervention subgroup analysis includes ear acupuncture, electroacupuncture, moxibustion, acupressure, traditional hand acupuncture, etc. The ultimate objective of this study is to hope that the conclusions drawn from the analysis can provide more useful evidence for the clinical operation and clinical research of acupuncture treatment of perimenopausal symptoms.

## Author contributions

**Conceptualization:** Qiujun He.

**Data curation:** Qiujun He, Yajing Ren, Yanqiu Wang.

**Formal analysis:** Qiujun He, Yanqiu Wang, Feng Zhang.

**Methodology:** Qiujun He, Yajing Ren.

**Project administration:** Qiujun He.

**Supervision:** Sanyin Zhang, Peng Yang.

**Validation:** Qiujun He, Yanqiu Wang, Feng Zhang.

**Writing – original draft:** Qiujun He.

**Writing – review & editing:** Qiujun He, Yanqiu Wang.

Qiujun He orcid: 0000-0002-1679-9625.
